# Universal health insurance in Rwanda: major challenges and solutions for financial sustainability case study of Rwanda community-based health insurance part I

**DOI:** 10.11604/pamj.2020.37.55.20376

**Published:** 2020-09-14

**Authors:** Médard Nyandekwe, Manassé Nzayirambaho, Jean Baptiste Kakoma

**Affiliations:** 1 University of Rwanda, College of Medicine and Health Sciences, School of Public Health, Kigali, Rwanda,; 2 Schools of Medicine and Public Health, University of Lubumbashi, Lubumbashi, Democratic Republic of the Congo

**Keywords:** Rwanda, community-based health insurance, financial sustainability

## Abstract

**Introduction:**

Universal Health Coverage (UHC) has engaged attention of policy makers at both global and country levels. UHC is one of three strategic priorities of World Health Organization's (WHO) general program of work for 2019-2023, and it is then a global health priority. Rwanda Community-Based Health Insurance is considered the vehicle for UHC and Universal Health Insurance in Rwanda. CBHI was officially introduced in 1999/2000 and through 2011/2012 Rwanda was not far from effective UHC. However, since then, CBHI faced chronic financial deficit. This study aims to assess challenges facing Community-Based Health Insurance financial sustainability and to propose indicative solutions.

**Methods:**

quantitative, qualitative, analytical, longitudinal (2011-2018) and documentary mixed methods were applied. One National Pooling Risk (100%), 15 Community-Based Health Insurance districts (50%) and 60 Community Based Health Insurance sections (13.33%) were randomly selected and included in the study. To assess major challenges, “analyzing qualitative data G3658-6 approach” and “prioritization hanlon method” were used.

**Results:**

the study highlighted five major challenges: (i) disproportionate risk-equalization in the social health insurance contributory system; (ii) unit cost exceeding individual income (premium plus other revenues and subsidies); (iii) imperfection in funding mobilization and recovery; (iv) cost-escalation; (v) diseconomy of scale; and the study proposed indicative solutions including injection of additional funding and shifting from current fee-for-service payment to fully active strategic purchasing mechanisms as accompanying measures.

**Conclusion:**

CBHI financial sustainability is achievable, but this is contingent upon persistence of political commitment efforts to achieve UHC, correction of highlighted imperfections and injection of additional funding to allow Rwanda Community-Based Health Insurance to meet and/or exceed its cost in the long-term.

## Introduction

Universal Health Coverage (UHC) has engaged the attention of policy makers at both global and individual countries levels [1]. The so-called World Health Organization (WHO), “UHC CUBE” conceptualized UHC as a process with three broad dimensions according to WHO 2010 [2,3]: the range of services that are covered (service coverage); the proportion of the total costs covered through insurance or other risk pooling mechanisms (financial coverage) and the proportion of the population covered. According to the UHC indicators for 2030 Sustainable Development Goals (SDGs), the target 3.8 of the 2030 SDGs comprises two metrics to measure progress towards UHC achievement, namely: indicator 3.8.1 “coverage of essential health services” and 3.8.2 “proportion of population with large household expenditures on health as a share of total household expenditures or incomes” [4]. The literature review showed that the process to achieve the UHC is a long journey. For instance, it took more than a century to pioneers industrial and/or developed countries [5, 6]. For Rwanda, the journey to move towards current UHC level was very short. Indeed, since 2000, Rwanda has developed the long-term strategy Vision 2020 whose health-goal is providing sufficient financial resources for universal access to quality health care to all Rwandan citizens at horizon 2020 [7]. To achieve this goal, Rwanda introduced “mutuelles de santé, now named Community-Based Health Insurance (CBHI) since 1999/2000, which is now available to all non-insured citizens, especially of rural population and informal sector [8-17]. Therefore, the Rwanda CBHI is considered a vehicle for UHC and Universal Health Insurance (UHI) in Rwanda. Between 2009 and 2011/2012, Rwanda was not far from effective UHC [2,9-16,18,19]. According to Nyandekwe M *et al*. the dream of effective UHC in Rwanda became a reality [20]; and according to Chemouni B. [9] and to Sanogo NA *et al*. [21], Rwanda is the most advanced country in Africa regarding UHC. As for 2019, most of UHC prerequisites have been achieved in Rwanda [20, 21], except some persisting gaps, for instance poverty and stunting among others [22-24].

Despite the impressive performance registered in Rwanda concerning UHC and (UHI), the CBHI financial deficit has become chronic from 2011/2012 until now. Indeed, retrieved deficit recorded in 2011/2012 was 3,896 Rwandan Francs (RWF) million (US$1=914.719 RWF, Exchange Rate July 29,2019) and 16,149 RWF million in 2014/2015 before it slightly declined to 12,837 RWF million in 2015/2016 [25], and jumped once again to 17,670 RWF million in 2017/2018 [26]. As a result, every year the Government of Rwanda (GoR) is obliged to intervene in the payment of CBHI debts to health facilities and this raises the question of whether the Rwanda CBHI is financially sustainable. Therefore, there is an urgent need to assure financial sustainability by identifying indicative alternative funding mechanisms and accompanying measures to support them strategically. The literature review showed also that bundled payments methods such as capitation, diagnosis-related groups (DRG) also called per-case payment (per visit, per admission, bed-day and/or inpatient stay-based) are considered active strategic purchasing models; they are recommended health services payment models employed to achieve health policy objectives in different countries [1,27]. These methods are deemed easy to administer and to forecast costs. They are also deemed as powerful cost-containment mechanisms and tools to improve the management efficiency [1,28,29]. Whereas numerous reports and publications on CBHI indicated that its performance was gradually remarkable, very few focused on its financial sustainability [20,26,30-32], and none has provided the long-term solution. This study aims to assess challenges facing Community-Based Health Insurance financial sustainability and to propose indicative solutions. The objectives of the study were: to identify challenges and gaps facing CBHI financial sustainability since July 1^st^, 2011 to date and to suggest indicative solutions to overcome major challenges and gaps related to CBHI financial sustainability in the strategic way.

## Methods

Quantitative and qualitative, analytical, longitudinal and documentary mixed methods were applied to explore and analyze primary and secondary data for the period 2011/2012 to 2015/2016 and beyond up to 2018 when data were available. The study was carried out countrywide, covering Kigali City and the four provinces. A stratified simple random sampling technique was performed from the “Cellule Technique d'Appui aux Mutuelles de Santé/CTAMS” 2014 database; one and unique Risk Pooling (100%) was automatically selected. Then, a simple random sample was done using 2011/2012 CBHI coverage status for 30 CBHI districts to select 15 CBHI districts (50%). The 2^nd^ simple random sample was done using the same 2011/2012 CBHI coverage status to select 60 CBHI sections out of 450 (13.33%) eligible i.e. operational at least since two years as inclusion criteria. After data cleaning, the study excluded one CBHI district and nine CBHI sections which had not correctly completed questionnaires for one or both fiscal years (FY) 2011/2012 and 2012/2013. The geographic distribution of CBHI entities finally retained in the study was as follows: one and unique Risk Pooling at national level and fourteen (46.66%) CBHI districts from which 1(7.1%), 3(21.4%), 2(14.3%), 6(42.9%) and 2(14.3%) were located respectively in Kigali City, Eastern Province, Northern Province, Southern Province and Western Province. Fifty-one (11.33%) CBHI sections from which 5(9.8%), 12(23.5%), 11(21.6%), 11(21.6%), 12(21.6%) were located respectively in Kigali City, Eastern Province, Northern Province, Southern Province and Western Province.

**Data analysis:** the study applied two methods commonly used in analysis of community health problems: the first is the 100% qualitative “analyzing qualitative data G3658-6” approach [33], used to: a) categorize CBHI financial sustainability-related challenges as per main CBHI functions, namely: social mobilization and sensitization, enrolment, collecting premium and legal contributions recovery, pooled funds and resources management, provider contracting and claims payment processing from various sources i.e. primary data and secondary data; and b) to sub-categorize the root cause(s). The second method is the prioritization Hanlon Method also known as the Basic Priority Rating System [34]. As every major challenge and deficit is quantitative, the severity was calculated as the relative importance of the ultimate challenge i.e. the overall deficit in 2015/2016. If the size was between 100% and 80.01%, the severity was scored between 10 and 8 according to its proximity to 100% or to 80.01%; if the size was between 80% and 60.01%, the score was 8 or 6; if the size was between 60% and 40.01%, the score was 6 or 4; if the size was between 40% and 20.01%, the score was 4 or 2; and if the size was between 20% and 0.01%, the score was 2 or 0. The CBHI and UHC performance were assessed using seven disaggregated UHC metrics developed by Stuckler D *et al*. [35].

### Concept and operational definition

**Cost recovery ratio:** the cost recovery ratio (CRR) is the ratio of total revenue to total operating costs, and is a key indicator of financial performance. It may be most conveniently expressed as a percentage. If it equals 100%, the operation as a whole is breaking even; if it exceeds 100% it is earning a surplus, while if it is below 100% the operation is losing money [36].

**Ethical considerations:** ethical approval was obtained from the Institutional Review Board of the University of Rwanda, College of Medicine and Health Sciences, School of Public Health; and the authorization of research was provided by the Rwanda Ministry of Health. All participants signed informed consent form prior to their enrolment.

## Results

### Major challenges, gaps and proposed solutions

**Challenge N°1: disproportionate risk-equalization in the UHI contributory system. Cause:** the risk equalization in the contributory system of CBHI was found to be disproportionate by the current study. Indeed, for civil servants, the GoR's subsidy allocated to their health insurance institution i.e. Rwanda Social Security Board (RSSB) Medical Branch was worth 7.5% of basic salary as a monthly subsidy paid by the GoR as matching subsidy while the civil servant himself paid the same proportion. For Military Medical Insurance (MMI) beneficiaries i.e. soldiers, policemen, Rwanda Correctional Service (RCS) personnel, the GoR paid 17.5% of the individual monthly basic salary to MMI while the beneficiaries paid a symbolic 5% of individual monthly basic salary. Calculated from a minimum of matching subsidy worth 3,000 RWF per CBHI beneficiary and for a total 7,906,824 CBHI beneficiaries during Year 2015/2016, the equivalent subsidy should have amounted 23,720 RWF million, equivalent to 185% of the 2015/2016 deficit estimated at 12,83737 RWF million; and then this major challenge was ranked first by the study. This means that the financial deficit could have been avoided if the adjusted matching subsidy (3,000 RWF) had not been halted from June 30^th^ 2011.This first major challenge was rated 10 because its magnitude was greater than 100% of CBHI deficit in 2015/2016.

**Proposed solution:** to reinstitute the matching grant at its current (adjusted) level worth 3,000 RWF that tripled for CBHI members from July 1^st^, 2011 to the present day versus 1000 RWF allocated to CBHI for every CBHI beneficiary from 2006 as defined at the beginning [37].

**Challenge N°2: incompleteness in legal allocations' mobilization and recovery. Causes:** (i) deletion of 13% of Ministry of Health (MOH) budget disbursed annually from 2006 as contribution to support the CBHI financial viability and halted on July 1st, 2011. Calculated from the 13% MOH in 2015/2016, it is equivalent to 18,712 RWF million, equivalent to 146% of the 2015/2016 CBHI deficit; (ii) this major challenge was ranked second by the study and was also rated 10 because its magnitude was greater than 100% of CBHI deficit in 2015/2016. Non effective recovery of the 5% gross premium cross-subsidy from public and private health insurance institutions, which is currently effectively recovered since 2015/2016; (iii) non adoption and non-implementation of 2010 CBHI Policy indicative contributions to CBHI financial viability, namely: 1% of domestic budget and, if necessary 1% of national budget, 1% annual revenue on tobacco, alcohol and other sin tax-based revenues.

**Proposed solutions:** (i) to reinstitute the 13% of MOH budget; if not to remove existing confusing terminology of 13% MOH appellation from the RSSB/CBHI budget, issue already solved for cross-subsidy recovery; and the adoption and implementation of the 2010 New CBHI Policy's indications. The internal mobilization of additional financial resources being extended to industries and factories with high health risk, to common heritage and to common natural resources, wealthy households belonging to the National Income Categorization Database (NICD) also referred to as “UBUDEHE” Category IV, not affiliated with RSSB, meanwhile, should pay an individual risk-sharing contribution worth RWF 3000 as proxy-contribution to cover unless one relative's health care pre-payment; while those beneficiaries of RSSB worth 8% of population [38] will contribute on the basis of their salary. With regard to external financing, it is urgent for Development Partners (DPs) to sign agreement on their annual financial commitment of the same minimum percentage for each of them.

**Challenge N°3: mismatch between unit cost and unit individual overall unit income per CBHI beneficiary. Cause:** overall unit cost exceeds overall unit income i.e. premium plus other unit revenues and subsidies. In Fiscal Year (FY) 2015/2016, other revenues included, the financial gap equaled 12,737,893,464 RWF (1611*9,680,581 RWF) and its magnitude was estimated at 99.23% (12,737,893,464 RWF *100/12.836, 594,044 RWF) of the 2015/2016 CBHI deficit, and thus rated 9.9 because its magnitude equaled 99.23% of CBHI deficit in 2015/2016.

**Proposed solution:** in the absence of the subsidies above mentioned reinstitution, mobilize substantial additional fund [s] internally as well as externally as proposed for challenge No2 above.

**Challenge N°4: cost escalation. Cause:** Fee-For-Service (FFS) provider payment medical claims equal to 17,007 RWF million, 22,505 RWF million, 26,069 RWF million, 36,307 RWF million, 34,183 RWF million, 35,6745 RWF million and 41,765 RWF million were registered for 2011/2012, 2012/2013, 2013/2014, 2014/2015, 2015/2016, 2016/2017 and 2017/2018, respectively. The in-depth analysis confronting medical claims, CBHI beneficiaries and health facilities utilization showed that the cost escalation could be the main cause of the difference of 5,707 RWF million revealed as abnormal increase occurred between 2013/2014 and 2014/2015. In fact, if the increase was normal, the Year 2014/2015 would have registered 30,600 RWF million instead of 36,307 RWF million. This fourth major challenge accounted for 45% (5,707 RWF million*100/12,737 RWF million) of the 2015/2016 CBHI deficit; so, the fourth major challenge was rated 4.5 because its magnitude equaled 45% of CBHI deficit in 2015/2016.

**Proposed solutions:** (i) to review the purchasing policy, ie to move from FFS provider payment mechanism (PPM) to move from FFS provider payment mechanism (PPM) to bundled payment methods, namely: capitation providers' payment at PHC level and to DRG-based provider payment at district hospital, specialized services, provincial hospital and referral hospital levels. (ii) Besides, it will require to introducing other cost-containment measures and/or management efficiency tools in the scheme.

**Challenge N°5: diseconomy of scale in CBHI system. Cause:** the continuous seeking of health-related universalism compliance. Although the latter is a priority in health for Rwanda, as the unit cost exceeds individual premiums, new enrollees including foreigner members from neighboring countries should not be welcome. This aggravates the existing financial gap in the scheme. In order to gradually comply with the health-related universalism in terms of CBHI's target population from 2016 to 2021, there is a need of integrating a portion of 5% (4.74%) poor and/or vulnerable people every year during the five following years (postponed to period 2019/2020-2023/2024) in order to achieve 100% of target in terms of covered population. Calculated on the basis of the 2015/2016 reference premium (5,019 RWF), i.e. the overall unit medical expenses plus the unit overhead expenses, this challenge could amount 2,429 341 802 RWF to be mobilized annually in order to cover related costs. The challenge represented 19% (almost 20%) of the 2015/2016 deficit, and thus it was rated 2 (1.9) because its magnitude was close to 20%.

**Proposed solutions:** (i) to formalize new additional funding mechanisms, which should complement existing contribution premiums and various legal subsidies to fill gaps without increasing individual premiums; (ii) to terminate enrollment of foreigners, prior to harmonization of the corresponding mutual reimbursement modalities between East African Countries (EAC) and the Democratic Republic of Congo (DRC); It should be noted that the DRC, and it could soon join the EAC; (iii) to set an optional contribution premium for foreigners, in line with Law N° 62/2007 of December 30^th^ 2007, asking them to be insured once their stay in Rwanda exceeds 15 days.

### CBHI and UHC performance from 2003 to 2017/2018 and current gaps

The Stuckler D *et al*. metrics [35] below were used since 2014 in the interim to critically assess Rwanda CBHI and UHC performance.

**Health insurance coverage:** since 2011/2012, the indicator increased from 7% in year 2003 to 91% in 2011/2012, dropped to 74% in 2013/2014 [25], increased to 82% in 2015/2016 and 84% in 2016/2017, and decreased a bit to 83% in 2017/2018 [26] versus 73.9% and 74% reported by 2013/2014 Fifth Integrated Household Living Conditions Survey 4 (EICV4) 2013/2014 and 2016/2017 EICV5, respectively [24]. Calculated from CBHI target population, the gap was 26%, 18%, 16% and 17% through 2013/2014, 2015/2016, 2016/2017 and 2017/2018, respectively.

**Access health services as Primary Health Care (PHC) utilization per person per year:** the indicator increased from 0.31 in 2003 to 1.72 in 2015/2016, decreased a bit to 1.65 in 2016/2017, increased to 1.94 in 2017/2018 patient visits at PHC level per capita per year [26] versus 1 visit recommended by WHO for Low-Income Countries. In the Organization for Economic Co-operation and Development (OECD) settings, the similar indicator reached 7.1 visits per capita in Year 2013 [39]. It is worth noting that the proportions of health services accounted for by CBHI are 95.8%, 97.6% and 99.3% in FY periods 2015/2016, 2016/2017 and 2017/2018, respectively [24]; and in general, 57% of Rwandan population who reported a health problem in the four week prior the EICV5 had medical consultation [24].

**Birth attendance:** this is a Sustainable Development Goals (SDGs) indicator (Indicator 3.1.2) considered as a proxy indicator of poor and/or utilization of health care. In Rwanda, the indicator increased from 35% in 2000 to 90.7% in 2015 [40] versus the basic target set at 75% by 2015 which is now surpassed.

**Equity in access to health services:** equity is demonstrated by the fact that one out of four (24.8%) persons of the Rwandan indigent are enrolled in CBHI system versus 24.1% in extreme poverty according to 2010/2011 EICV3[41], 22.8% indigent people were enrolled versus 16,3% in extreme poverty according to 2013/2014 EICV4[23]; and 17.1% indigent people were enrolled versus 16% in extreme poverty according to 2016/2017 EICV5 [24] respectively. However, some people belonging to UBUDEHE lower category II are in need of subsidy to enroll CBHI [42].

**Rights-based approach:** CBHI beneficiaries have the same right to comprehensive quality health care, from community to tertiary level in a roaming system manner; but this is subject to a mandatory reference from the first health center used. However, as mentioned above in the area of equity, iniquity to persons deprived of access and other undetermined social economic and social cultural determinants would not allow the authors to certify the full right to health care of all CBHI beneficiaries.

**Package of services:** CBHI beneficiaries have access to entire domestic package health care provision from community package to tertiary package within all contracted public and FBOs health facilities (private non-for-profit) health facilities. However, entire package health care provision is not provided to CBHI beneficiaries; the gap includes, among others: community-based eye care screening, eyeglasses and other essential eye care services. Added to these are the prostheses, ortheses, magnetic resonance imaging (MRI), computed/computerized tomography scan (CT-Scan) and dialysis.

**Quality of health care:** according to numerous authors, the quality of health care services should be good enough to improve the health of those receiving services [43-45]; this is in line with Quality Assurance Strategy and with Performance Based Financing now linked with accreditation. In 2011, the client satisfaction of health services was quantitatively estimated at 92% [46] versus 89% in 2013/2014 (EICV4) and 85% in 2016/2017 (EICV5) [24]. However, poor medical attention and delays in transfers to referral hospitals were reported by medical practitioners and members of the scheme as major issues that need to be resolved [38].

**Catastrophic Health Expenditure (CHE):** the catastrophic health expenditure faced by Rwandan people was estimated at 11.9% in 2000 and at 7.7% in 2006 in the total population [17]; the same indicator level equaled 10.80% versus ≤ 40% as acceptable limit level in 2011/2012 [20], and decreased to 8% and 7% according to the EICV4 [23] and EICV5 [24], respectively. Regarding indicator on health care paid collectively, a symbolic flat 200RWF is co-paid at health center by non-poor client as deductibles; while, at hospitals levels, 90% of billable health care services are paid collectively through CBHI central Risk Pooling and 10% are co-paid by non-poor patients; this demonstrates a good protection of CBHI beneficiaries against financial hardship at the point of use. However, the 10% co-payment is found unaffordable by the majority of Rwandan population. For instance, 44,1% of all Rwandan people faced unless one health chock and used savings or borrowing to receive health care [24]. As a positive impact, remarkable health gains are reported, for instance: life expectancy increased from 49.71 years in 2001 to 66.6 years in 2016/2017 [24] versus 55 years as initial target set for 2020 [7]; all SDGs targets are achieved except prevalence of poverty worth 38.1% in 2017 versus 30.2% as related target for the Millennium Development Goals (MDGs) 2015 [24]; and stunting worth 38% in 2014/2015 versus 24.5% as related target for MDGs 2015 [40].

**Figure 1:**
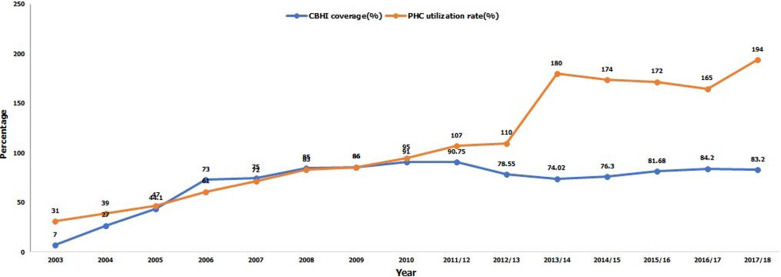
trend of CBHI coverage (%) and PHC utilization rate (%) from 2003 to 2017/2018

**Cost recovery ratio:** Cost recovery ratio: the indicator was estimated at 82.96% in 2011/2012 according to the RSSB/CBHI Report 2015/2016 [25] versus 108.69% in 2011/2012 according to Nyandekwe M. et al. 2014 [20] and worsened to 67.65% in 2015/2016 [25], increased a bit to 70.13% and declined again to 63.29% [25] for 2016/2017 according to RSSB/CBHI FY 2016/2017 Report [31] and 2017/2018 according to RSSB/CBHI FY 2017/2018 Report [26], respectively. The related financial gaps of 32.35%, 29.87% and 36.71% were to be filled through the same respective fiscal years, revealing that additional substantial funds are urgently needed (Figure 1).

## Discussion

### Major challenges and proposed solutions

#### The top four challenges are discussed per respective size decreasing order

**Disproportionate risk-equalization in the social health insurance contributory system:** concerning equity in the broader sense, the GoR put in place a good risk-sharing mechanism. However, the equity in the contributory system between CBHI and other social/public health insurance schemes was found disproportionate. In fact, what could happen to the financial sustainability of the public and social health insurance schemes if the subsidy from the GoR is removed? Similarly, what would really happen if the majority of Rwanda CBHI target population (almost 94% of the population) would ask for the full right to use private health facilities and related social protection long-term benefits provided to civil servants, soldiers, policemen and RCS personnel? The authors find that it is reasonable to provide CBHI with additional substantial funding to guaranty that its financial sustainability would be comfortable in the absence of corresponding long-term social benefits.

**Remaining top three challenges:** with regard to the remaining top three challenges, namely (i) “incompleteness in mobilization and recovery of legal allocations” promised by the GoR and Development Partners, (ii) “non-adoption and non- implementation of 2010 CBHI Policy indications” and (iii) “mismatch between unit cost and unit income per CBHI beneficiary”, the GoR should be aware of it and promptly contribute accordingly; the health-related universalism goal by 2020 requires it. With particular regard to the abolition of 13% MOH budget allocation since the implementation of 2010 CBHI Policy, the continuous allocation of indigent enrollment subsidy under the same budget line item is surprising. Indeed, the budget line item still appears within the RSSB Strategic Budget for 2016-2019, but related amount is rather the subsidy for the needy, and therefore this erroneous appellation should change.

**Literature review versus study results:** with regard to the proposed accompanying measures, i.e. the active strategic purchasing mechanism, this is not new in Rwanda. In fact, the three required steps were reached by Rwanda since the large-scale of CBHI in 2006, namely: (i) there is a defined minimum package of activity from community level to tertiary level as benefit packages to CBHI beneficiaries including listed essential medical procedures and medicines authorized by level; (ii) the tariffs setting system offers to CBHI beneficiaries a minimal tariff compared to other health insurance schemes, and (iii) CBHI contracts with all public and faith-based organizations (FBOs) health facilities including the one private referral King Faisal Hospital (KFH) and some private health posts at primary health care level. However, the existing strategic purchasing mechanism efficiency is inhibited by the FFS payment mechanism practice, which is still considered “passive” by the study. Therefore, the “active” character in the purchasing system is avidly expected to be implemented in order to protect the awaited substantial funding against cost escalation encouraged by the FFS practice.

**Study limits:** some bundled payment models do not always avoid cost escalation; for instance, the bed day and inpatient stay-based models may encourage providers to extend the stay of patients, on the one hand; while most of these models may lead to under-provision of health services, on another hand [29, 47]. Other additional gate keepers' measures are then needed to fight against cost escalation and to minimize the low quality health care provision. The current study has not polled opinions from Rwandan health providers about their claims and expectations regarding FFS, capitation, DRG and other bundled methods. According to Hsiao WC *et al*. [5], in case of bundled payment mechanisms, health providers with higher costs would not want to contract with social health insurance unless they can bring down their cost to these established payment levels. Therefore, most health providers are anti-capitation and it is difficult to obtain unanimous agreement from them before implementing those models [2]. Even though the opinion of this stakeholders group has not been polled, the authors assume that impressive records from Rwanda CBHI from 2011/2012 to 2017/2018 and extensive evidence-based data from validated public documents should help decision-making process.

## Conclusion

The study shows that the top five main challenges could be overcome. However, this depends on the strong and persistent political commitment to effective UHC and correction of imperfections identified by this study. In order to address the financial sustainability of the CBHI and to improve health-related universalism outcomes, the authors recommend additional substantial funding. They also recommend moving from the current FFS as a passive strategic purchasing mechanism to bundled PPM models aiming at protecting future substantial funding against cost escalation.

### What is known about this topic

- In general, challenges facing CBHI since its first introduction in 1999/2000 to present were gradually known but few focused on financial sustainability and none treated solutions aspects.

### What this study adds

- This part I of the study identifies challenges facing CBHI financial sustainability, analyses their respective root causes and proposes indicatively long-term solutions;
- Part II of the study will serve as scientific evidence-based data to refining a basic quantitative study on Feasibility of CBHI financial sustainability.
